# Advances in Ghost Imaging of Moving Targets: A Review

**DOI:** 10.3390/biomimetics8050435

**Published:** 2023-09-19

**Authors:** Moudan Shi, Jie Cao, Huan Cui, Chang Zhou, Tianhua Zhao

**Affiliations:** 1 The School of Optics and Photonics, Beijing Institute of Technology, Beijing 100081, China; 13261993633@163.com (M.S.); sonya_cui@163.com (H.C.); 3120225338@bit.edu.cn (C.Z.); 3220220482@bit.edu.cn (T.Z.); 2 Yangtze Delta Region Academy, Beijing Institute of Technology, Jiaxing 314019, China

**Keywords:** ghost imaging, moving blur, correlation imaging, moving imaging

## Abstract

Ghost imaging is a novel imaging technique that utilizes the intensity correlation property of an optical field to retrieve information of the scene being measured. Due to the advantages of simple structure, high detection efficiency, etc., ghost imaging exhibits broad application prospects in the fields of space remote sensing, optical encryption transmission, medical imaging, and so on. At present, ghost imaging is gradually developing toward practicality, in which ghost imaging of moving targets is becoming a much-needed breakthrough link. At this stage, we can improve the imaging speed and improve the imaging quality to seek a more optimized ghost imaging scheme for moving targets. Based on the principle of moving target ghost imaging, this review summarizes and compares the existing methods for ghost imaging of moving targets. It also discusses the research direction and the technical challenges at the current stage to provide references for further promotion of the instantiation of ghost imaging applications.

## 1. Introduction

Ghost imaging (GI), also known as correlation imaging, differs significantly from conventional imaging in localized nature. In conventional imaging, the target image information is obtained by recording the light intensity distribution of the radiation field with focal plane arrays [1,2], while GI records the light intensity values with a barrel detector that does not have spatial resolution [3,4] and computes the correlation with the modulation patterns to reconstruct the image of the target object. To simplify the GI system, researchers have proposed computational ghost imaging (CGI) [5] and single-pixel imaging (SPI) [4,6,7], which require only one optical path to achieve image reconstruction of the target object. The resolution limit is related to the coherent intensity of the optical field, and the image resolution can exceed the Rayleigh diffraction limit, while the application of compressive perception theory [8] to GI enables low Nyquist frequency sampling. Numerous advantages of GI make it promising for applications in space remote sensing [9], optical encryption [10,11], LiDAR [12,13,14], medical imaging [15,16,17], near-infrared imaging [18], terahertz imaging [19,20], broadband and hyperspectral imaging [21], X-ray imaging [22,23,24], and imaging of scattering media [25,26,27].

Many practical applications are inseparable from the imaging of moving objects, but the relative motion between the object and the imaging system will inevitably affect the imaging. For traditional imaging, the elative motion between the object and the imaging system causes the resolution of the image to decrease. GI requires multiple measurements to reconstruct the object image, and the relative motion between the object and the imaging system also causes motion blur and increases the difficulty of imaging. Imaging of moving objects is an important part of GI towards practical use, such as LiDAR, space remote sensing, security monitoring, autonomous driving, etc. At present, how to improve the performance of moving target ghost imaging is one of the key problems to be solved. The purpose of this review is to summarize the research and applications of GI technology for moving targets, and analyze the key problems and development trends of GI for moving targets, providing references for in-depth research of this technology.

## 2. Theoretical Basis of GI

GI achieves reconstruction of target characteristic parameters by correlating the two-dimensional light field intensity distribution of the light source and the total light intensity value of the echo light field carrying the target modulation information. Through the introduction of light field fluctuation modulation and computational reconstruction, GI not only has higher information acquisition efficiency, but also has improved flexibility of image information acquisition. The basic idea of GI can be traced back to the Hanbury Brown–Twiss (HBT) experiment in 1956 [28], which enabled the measurement of the angular diameter of a star by correlating the intensity correlation of the probe light. Early GI used an entangled light source, so its image mechanism was considered to be a quantum entanglement effect. However, Boyd et al. [29] reproduced GI using a classical light source in 2002, the first theoretical analysis and experimental verification of the feasibility of classical light fields for GI experiments; generally common GI are two-arm conventional GI [30,31], CGI [29,32,33,34] and SPI [34,35,36].

**Figure 1:**
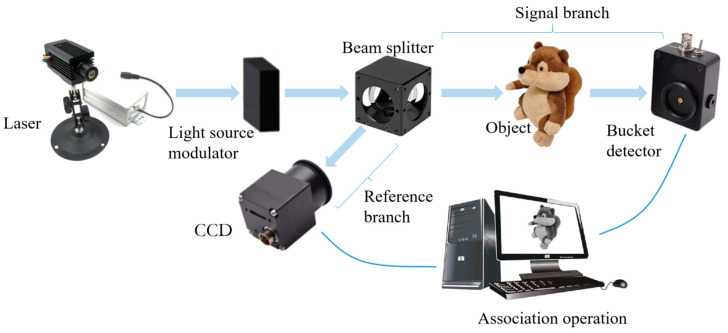
Schematic diagram of a two-arm conventional GI system.

The two-arm conventional GI system has two branches, shown in Figure 1. The light from the laser is passed through the light source modulator to obtain the scattered light field for GI, after the beam splitter is divided into a signal branch and a reference branch. The barrel detector in the signal branch receives the reflected or refracted light from the target object and records a light intensity value. The reference branch uses a charge-coupled device to collect the light field information. The image of the target object is reconstructed by associating the light intensity information of the two branches [37].

**Figure 2:**
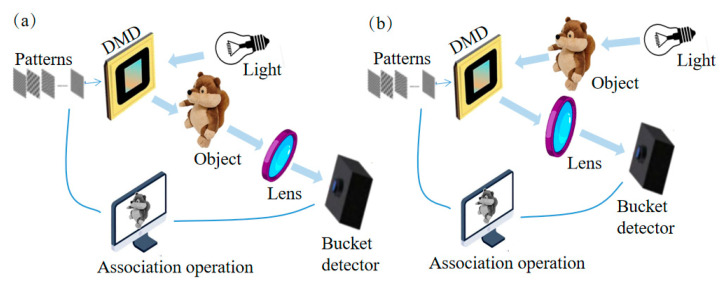
Principle diagram. (**a**) Principle diagram of CGI. (**b**) Principle diagram of SPI.

To simplify the GI system, a CGI method was proposed by Shapiro [5] in 2008 and Bromberg [3] experimentally validated CGI in the following year. CGI is an indirect imaging method that uses a single pixel photodetector without spatial resolution to reconstruct the image of a target object type. Unlike two-arm conventional GI, CGI has only one branch, as shown in Figure 2a. The key to modulate the scattered light field is to use a digital micromirror device (DMD) or a spatial light modulator (SLM) [5], then to rely on a bucket detector with no spatial resolution to receive light intensity information. CGI can improve image quality by designing scatter patterns, which has advantages over two-arm conventional GI [34].

There is another optical path design based on spatial light modulation for GI, as shown in Figure 2b, where the light beam from the light source is reflected or refracted by the target object [4,6,7] and modulated by a DMD. Then, the resulting light field is detected by a single-pixel detector, and the image of the target object is reconstructed by correlating the light intensity value with the modulation patterns, which is the SPI proposed by Baraniuk et al. [4,6,7] in 2008. SPI is a new imaging method that uses a single pixel detector and an SLM to obtain images through reconstruction. Compared with traditional array detector imaging, SPI has the advantages of high sensitivity and anti-interference, and has a very broad application prospect in many fields [38].

In 2008, CGI and SPI concepts were proposed almost simultaneously. From the perspective of technology development history, CGI and SPI are two independently developed technologies, but their theoretical basis, implementation methods, and reconstruction algorithms have many similarities. From an optical point of view, CGI and SPI are essentially the same; the only difference is the order of the SLM (or DMD) and the image object in the light path. In the CGI optical path, the way that light is modulated through SLM and then illuminated to the object is called structural lighting [34]. In the SPI optical path, the light passes through the imaging object and is modulated by an SLM, which is called structured detection [38]. Nowadays, CGI and SPI are completely universal in imaging principle, modulation strategy, and reconstruction methods [6].

## 3. Research Status of Moving Target GI

Moving target GI differs from stationary target GI in that it has higher requirements for temporal and spatial resolution. The typical problems common to moving target GI are twofold: the limited processing speed of the image system leading to the inability to image in real time, and the image blurring problem caused by the relative moving between the object and the image system. In conventional imaging, moving blur occurs if the object moves on a light-sensitive surface at a distance larger than the pixel size during the exposure time of the camera; a shorter exposure time is usually used to solve this problem. In GI, the relative moving between the target object and the optical axis causes the lateral resolution of the image reconstructed by GI to deteriorate, which produces moving blur, as demonstrated theoretically and experimentally by Han et al. [39] who proved it both theoretically and experimentally in 2015. At present, most reviews of ghost imaging focus on stationary targets, and there have been few articles on the status of moving targets. This paper focuses on the existing problems of moving target GI and divides the existing research methods into two major parts: improving the image speed and the quality. To improve reconstructed image speed, researchers use six methods, namely, improving light source modulation methods, selecting the adaptive image region, selecting a suitable number of samples, estimating motion inter-frame information, developing new reconstruction algorithms, and tracking the target without image reconstruction. Designing novel modulation patterns and moving the compensation for modulation patterns are used to improve image quality. Researchers use these methods to reduce the image blur caused by the relative moving of the object and the image system, and to improve the image quality.

## 4. Improving Image Speed

Image speed is quantified by image time. The shorter the image time, the faster is the image speed. The image time of GI is the sum of the data acquisition time and the reconstructed image time [38], where the data acquisition time can be expressed as the ratio of the number of modulated scatter spots to the modulation rate. Therefore, this paper details below the contributions made by researchers in improving the image speed of GI of moving targets in the six aspects mentioned.

### 4.1. Improved Light Source Modulation Method

In GI, image information can be obtained and processed flexibly by designing the modulation mode of the light source. In early pseudo-thermal light source GI, a rotating piece of gross glass was placed in front of the light source to Gaussian modulate the light source [29]. With the advent of CGI, researchers began to modulate the light source using a projector, a spatial light modulator (SLM), and a digital micro-mirror device (DMD) [3]. Up to 60 patterns per second can be projected with the projector. Even the most advanced DMD modulation speeds can reach 22 kHz, but the speed drops dramatically when multi-gray patterns are produced [38].

**Figure 3:**
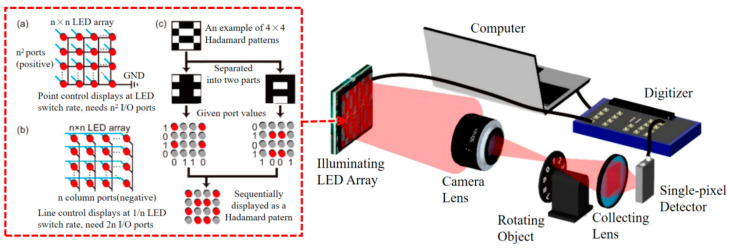
High-speed LED lighting module experimental schematic [41].

To improve the light source modulation speed, Song et al. [40] in 2016 utilized LCD to generate structured pseudo-random patterns with a size of 128 × 128 pixels. This method simplifies the control process of the light source, can greatly reduce the number of measurements required for image reconstruction, and can be clearly imitated even if there is an external light source. In 2018, Sun et al. [41] developed a 32 × 32 pixels high-speed LED illumination module, with a light field refresh rate up to 500 kHz; the schematic of which is shown in Figure 3, using Hadamard patterns as the modulation patterns. It was able to display them at half the LED switching rate, achieving a continuous image at a frame rate of 1000 Hz, about two orders of magnitude greater than other existing GI systems. The object they used was a black disc with ten numbers uniformly engraved from 0 to 9, which rotated at a specified speed, recorded light intensity information with a single-pixel detector, a data acquisition card that was synchronized with the LED array, and then transmitted the intensity data to a computer for image reconstruction. In the same year, a Spanish group [42] used an LED light source with a refresh rate of 10 kHz to achieve a 32 × 32 pixels 3D object image at a frame rate of 10 Hz, resulting in an SNR of 53 dB for color images and 62 dB for monochrome images. In 2019, Chen et al. [43] achieved a video image at a 1.4 MHz frame rate using an LED array light source with a refresh rate up to 100 MHz. The LED array works at a frame rate of 1 MHz, the imaging frame rate is changed to 5 kHz, the light intensity value is detected using a single-photon detector (SPD), and when the photon reaches the detector, the SPD generates an electronic pulse as a count. However, a single SPD has the problem of not being able to represent the number of photons and having a dead time, so an eight-mode SPD detection system was built. In this system, eight multi-mode fibers with a core diameter of 50 microns are tightly packed at one end, and their other end is connected to an SPD. The signal collected by the detector is fed into the time-correlated-single-photon-counter, and the arrival time of each signal can be obtained, thus obtaining the number of photons in each time window. In the same year, Inoue et al. [44] proposed the use of an optical correlator as a spatial modulator to acquire a reconstructed image using 1000 random binary patterns and obtained an image frame rate of 133.7 fps. By improving the modulation of the light source, the modulation speed of the light source is improved, and the time required for data acquisition is reduced, which is important for the early realization of real-time GI of moving objects.

### 4.2. Selecting the Adaptive Image Region

**Figure 4:**
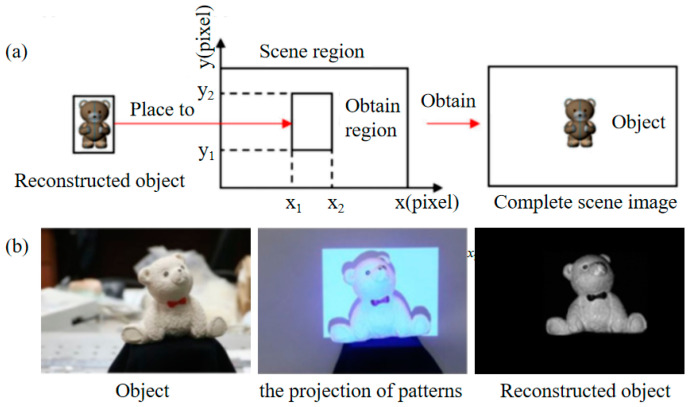
Adaptive area SPI method [45]. (**a**) Schematic representation of image reconstruction. The three images are the ARSI reconstructed ground object image, the localized target region, and the fully reconstructed image. (**b**) Experimental diagram of image reconstruction. The three images are, respectively, the target object, the localized object region illuminated by the digital projector, and the full reconstructed image [45].

Typically, the object to be imaged occupies only a fraction of the entire region, and if SPI is performed only for the target region, the number of modulated patterns can be significantly reduced without degrading the image quality. In 2017, Zhao et al. [45] proposed an adaptive region SPI method for the case where the object occupies part of the illuminated region; the schematic diagram is shown in Figure 4. This method obtains slices of Fourier coefficients by projecting vertical and horizontal two-dimensional sinusoidal patterns. On the projected line of the scene, however, the position of the object edge changes due to the different grayscale distribution of the object and background. Therefore, the Fourier slice theorem and the edge detection algorithm can be used to adaptively localize the target region, and the Fourier SPI method can be applied to reconstruct the target image for the target region. This then places the reconstructed image at the location of the object in the scene to generate the full image. It can greatly reduce the number of modulation patterns and improve the image speed.

### 4.3. Selecting a Suitable Number of Samples

Typically, moving targets are sparse, and vehicles traveling on roads have small scattering interfaces and spatial sparsity with respect to the surrounding buildings. Choosing the appropriate sampling number can effectively reduce the data acquisition time [38] and improve the image efficiency of GI.

**Figure 5:**
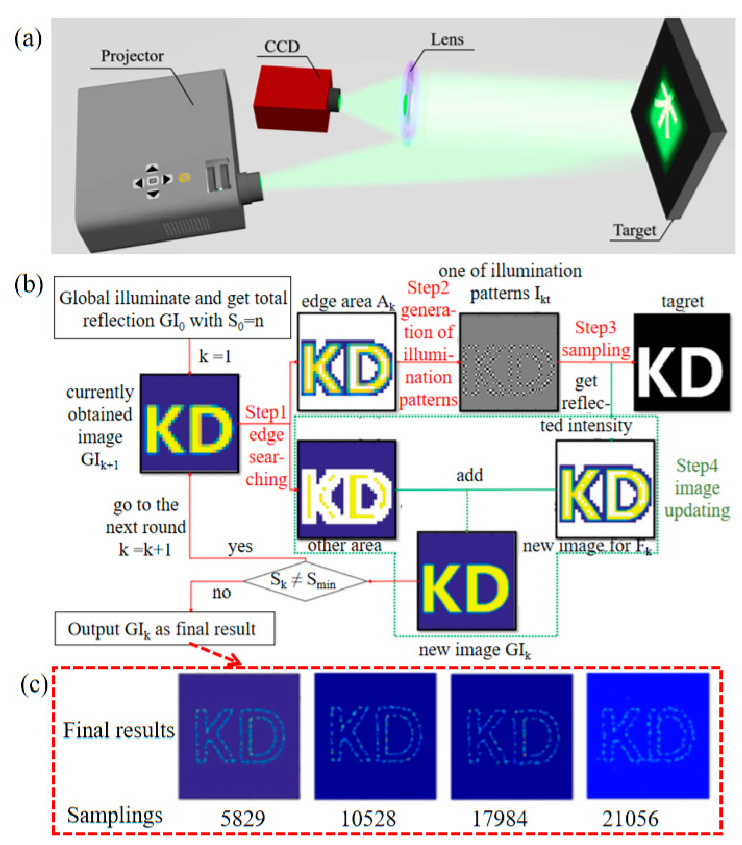
Feedback GI. (**a**) Schematic diagram of experimental apparatus [47]. (**b**) The entire scene is illuminated, and the reflected intensity of the measured target is GI_0_ with resolution S_0_ = n. The following four steps are then followed sequentially: edge search, sampling, generation of illumination pattern, and image update. The arrows show the steps and the direction of the data. The red arrow in Step 3 also indicates that the illumination pattern is illuminated onto the target. (**c**) The reconstructed images with sample rates of 5829, 10,528, 17,984, and 21,056.

In 2019, Liu et al. [46] proposed a temporal intensity difference correlation GI scheme, which exploits the spatial sparsity of moving targets and can acquire high-quality images of moving objects in complex scenes with fewer samples. It only requires a linear algorithm and significantly reduces the reconstruction time of the image, which is important for tracking. This method can handle the relative motion of multiple moving objects and remains effective even when the shape of the moving object changes. Experimentally, the tracking and imaging of two moving objects with different speeds and orientations was implemented. In 2021, feedback GI strategy to reduce the number of samples was proposed [47], shown in Figure 5. It adaptively adjusts the field-of-view and scatter size based on the image and concentrates the high-resolution scatter in the edge regions. It can extract more side information and requires a much smaller number of samples than regular GI due to the reduced field of view. Choosing the right number of samples can greatly reduce the time required for sampling, which correspondingly reduces the time for the correlation operations and improves the image speed of GI.

### 4.4. Estimating Moving Inter-Frame Information

The high-resolution tracking of moving objects can be achieved by associating each moment of a moving object with an associative operation and obtaining the shape and position information of the object at the corresponding moment through the different images of adjacent moments. However, this increases the data volume and time cost of the association operation. To solve this problem, researchers have divided the object moving into multiple moving frames and achieved GI of the target object through estimation of the information between moving frames.

**Figure 6:**
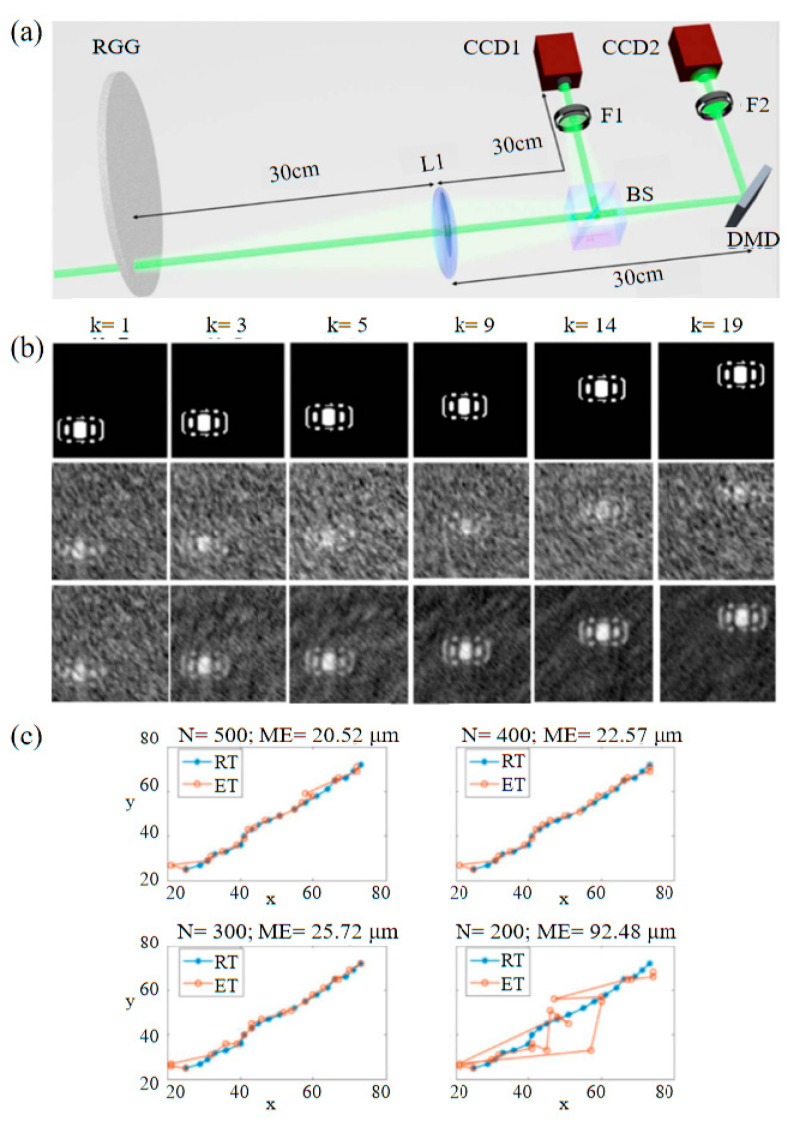
Moving objects by tracking based on cross correlation [48]. (**a**) Schematic diagram of experimental apparatus. Both CCD1 and DMD are located in the Fourier plane of lens L1. (**b**) Reconstructed images, where the first act is a car in a different location, the second act is a blurred image sampled 400 times per frame, and the third act is a CBGI reconstruction of the image. (**c**) Sample real trajectories and calculated trajectories for different times per frame. When the number of samples N is more than 300, there are good results, but when N is less than 200, the ME between RT and ET is larger than the resolution of the image, then the method will not work well.

In 2019, Liu et al. [48] proposed Gradual GI of moving objects by tracking based on cross correlation. The experimental setup and experimental results are shown in Figure 6, which uses less sampling to obtain blurred images of objects, calculates the image correlation to obtain the displacement of the object at the corresponding instant, and then gradually reconstructs a high-quality image during the object’s motion. The moving inter-frame information estimation reduces the amount of data in the multi-frame image transmission channel and improves the image speed. This method works well when 300 flash samples are taken per time frame, but it will not work well if the object is moving too fast and the number of samples per time frame is reduced to 200.

### 4.5. Developing New Reconstruction Algorithms

The GI obtains the reconstructed image of the target object through the reconstruction algorithm, so the computational efficiency of the reconstruction algorithm plays a decisive role in the image reconstruction time; advanced imaging algorithms can greatly reduce the number of samples required for imaging, which can improve the image speed. 

**Figure 7:**
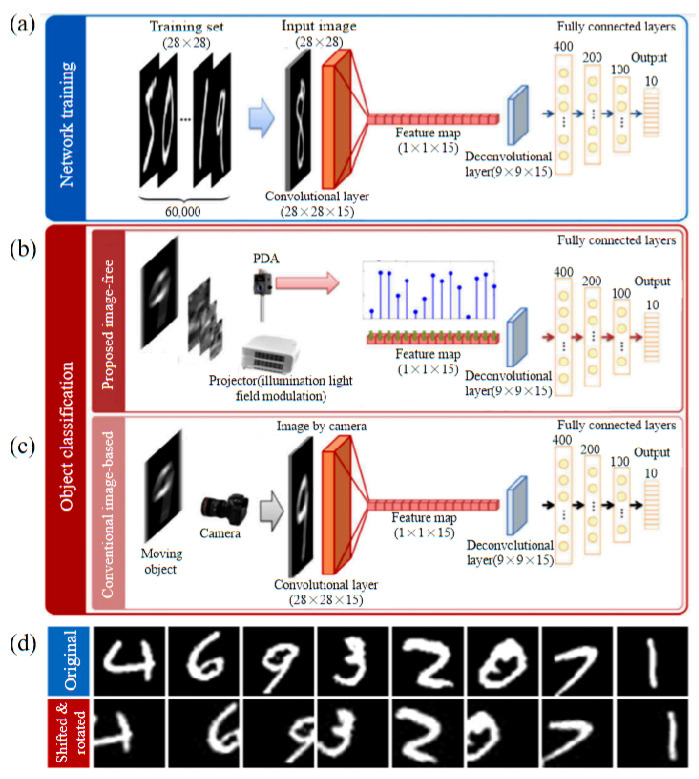
Target classification schematics based on CNN and examples of training images [50]. (**a**) Structure of the proposed CNN. Similar to traditional CNN, the CNN proposed in this paper consists of an image input layer, which accepts images as input during network training. The convolutional kernels in the trained CNN are used as patterns for structured illumination; different from traditional CNN, when the CNN proposed in this paper is used for object classification. (**b**) The feature map becomes the input layer that takes single-pixel measurements as input. A single pixel measurement is obtained by using a convolution kernel to illuminate a moving object. (**c**) This requires high-speed photography, and this approach achieves object classification without acquiring images. (**d**) The first row is the original image and the second row is the randomly shifted and rotated image laterally.

In 2021, Sagi Monin et al. [49] proposed an algorithm to estimate the moving between consecutive frames and integrate it into a model matrix for SPI. It improves the numerical efficiency of the algorithm by estimating the global motion of the target object from the measured data via a circular model matrix without any image reconstruction. They used this method to track and image the global motion and local motion of the object respectively. In the same year, Zhang et al. [50] proposed a real-time classification method for fast moving objects without image acquisition; the schematic diagram and simulation results are shown in Figure 7. The key point is to directly obtain the target features using structural illumination and single-pixel detection and train a convolutional neural network to learn the target features. It then feeds the single-pixel measurements into a trained convolutional neural network to achieve accurate and real-time classification of fast-moving objects. It can be performed in a 45 mm × 45 mm field of view and can successfully classify objects with a speed of 3.61 m/s. The achievable temporal resolution is 1.68 ms. Each classification requires only 1680 bytes of data. The computation time is 1.43 ms. This method is both data efficient and computationally efficient, allowing real-time and long-time classification.

### 4.6. Tracking Target without Image Reconstruction

Target tracking methods include image-based and image-free methods. Image-based tracking methods rely on continuous image acquisition and subsequent processing, and have low tracking efficiency, while image-free target tracking methods detect and track fast-moving objects in real time.

**Figure 8:**
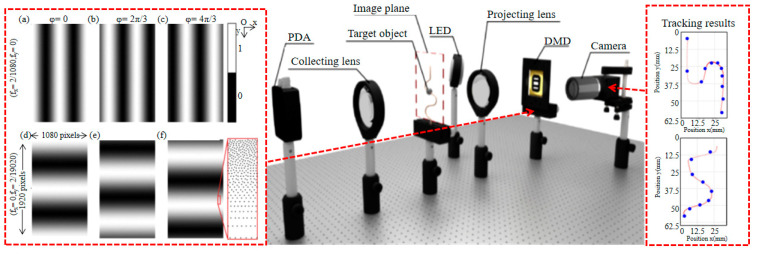
Real-time detection and tracking of fast-moving objects without images [52]. The picture on the left is of Fourier patterns, (**a**–**c**) for obtaining the light intensity value of the object on the *x*-axis, (**d**–**f**) for obtaining the light intensity value of the object on the *y*-axis.

In 2019, Shi et al. [51] proposed a fast target tracking technique based on SPI. The key point of this approach was to construct modulation information that satisfies the projection condition, transforming the 2D image into a 1D projected curve. The tracking of the moving target is achieved by acquiring the 1D projected curve of the moving target in real time with high accuracy, which provides the location information of the moving target. They also proposed a background subtraction technique for tracking moving objects, which can remove static components in the scene and speed up the tracking of SPI. They used this method to track moving objects with less than 0.2% of the measurements established by the Nyquist criterion, and it presents 256 × 256 pixels at ~177 fps. In the same year, Zhang et al. [52] proposed an image-free, real-time tracking method for fast moving objects, shown in Figure 8. They used six Fourier fundamental patterns for structured light modulation to measure only two Fourier coefficients in the complete Fourier spectrum of the object image. Then they used SLM and single-pixel detection to acquire spatial information of the target object, but not the image for target detection and tracking. A temporal resolution of 1/1666 s was achieved by using a 10,000 Hz DMD, but only a moving object which tracked in just two dimensions could be detected. The following year, this research group [53] implemented an image-free 3D tracking method. It used six single-cycle Fourier basis patterns for illuminating a moving target, and used only two single-pixel detectors and a high-speed SLM for data acquisition, then used the corresponding single-pixel measurements to resolve and calculate the position of the target. It can detect and track fast-moving targets at a frame rate of 1666 frames per second on a 10,000 Hz DMD. In 2022, Yu et al. [54] proposed an image-free real-time target tracking scheme which is based on discrete cosine transform and single-pixel detection. This approach uses complementary modulation to reduce measurement noise and background phase subtraction to enhance contrast. It can avoid the computation of all phase values and drastically reduce the number of samples. This method can track moving targets under a complex background, and the sampling rate is less than 0.59% of the Nyquist–Shannon criterion; the fastest tracking speed can reach 208 fps.

## 5. Improving Image Quality

The image quality of GI is inextricably related to the modulation patterns; the modulation sequence of the Hadamard matrix sequence has a significant effect on the image quality [55]; the resolution of the reconstructed image can be adjusted by controlling the transverse cohere length of patterns [17], and the contrast of the reconstructed image can be improved by using a scattered light field with super Rayleigh distribution [56]. For moving target GI, the design of new modulation patterns and the moving compensation of the modulation patterns can improve the image quality. 

### 5.1. Designing New Modulation Patterns

**Figure 9:**
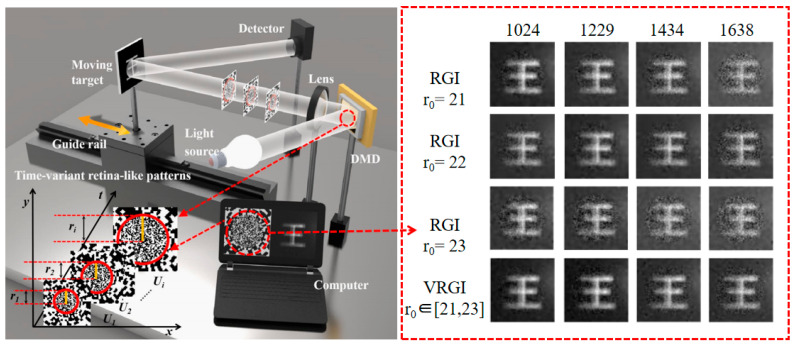
Schematic diagram of the experimental setup for time-varying retinal-like computational GI of axially moving targets [57]. The images on the right are RGI and VRGI reconstructed images of axially moving objects at sampling numbers 1024, 1229, 1434, and 1638. Here, RGI stands for the CGI method with retina-like patterns. The number of samples here refers to the number of patterns required to reconstruct an image. r0 in the figure is the radius of the fovea region.

**Figure 10:**
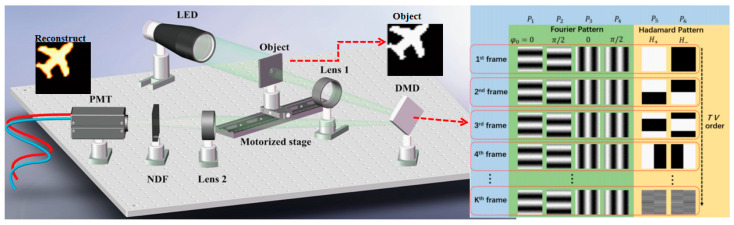
Schematic diagram of the experimental setup and pattern design [60]. On the right is the design of the pattern. Each moving frame corresponds to six patterns, four of which are binary Fourier based patterns and two are differential Hadamard patterns. The Fourier patterns of all frames are the same, and the corresponding phases are 0 and π/2 respectively. According to the total variation (TV) sorting method, the Hadamard patterns corresponding to different moving frames are sorted.

In 2022, our group [57] proposed a time-variant retina-like computational ghost imaging (VCGI) for axially moving targets; the schematic diagram of which is shown in Figure 9. It uses 64 × 64 pixels retinal-like patterns with a variable central concave region radius to reconstruct axially moving targets; the target moves evenly along the optical axis, and the total movement distance is 5 mm. It is worth mentioning that the radius of the central concave region can be modified according to the axial movement of the target. It provides good control of the light field during the movement of the target, resulting in high-quality reconstructed images. In the same year, Shi et al. [58] proposed a moving-compensated SPI method based on time-division multiplexing. It uses geometric moment patterns and Hadamard patterns to time-division multiplex the target position information and uses image information alternate encoding to localize moving objects at high frame rates. It improves the performance of moving blur-resistant SPI and meets the demand for SPI in more moving scenes without additional hardware to localize or estimate the moving state of the object. When the object angular velocity is as high as 0.5 rad/s, the positioning frame rate can reach 5.55 kHz, and the image of 512 × 512 pixels can be reconstructed. In the same year, Fu et al. [59] proposed an effective method for image random moving targets based on geometric moment analysis. Each frame was divided into 20 slices and the moving state of each slice could be obtained by using cake cut-order Hadamard patterns and low-order geometric moment patterns, to obtain high-quality video streams of targets moving at different translational and rotational speeds. This method can reconstruct a randomly moving object with a rotational speed of 1800 revolutions per minute. In the following year, Li et al. [60] proposed a method to obtain the relative displacements and images of translational objects simultaneously. It uses four binary Fourier patterns and two differential Hadamard patterns as shown in Figure 10 to modulate one frame of the target. The method does not require any a priori knowledge to obtain the relative displacement and image of the object, and the quality of the reconstructed image improves rapidly and stabilizes as the number of measured frames increases. The method achieves the relative displacement of the moving targets at 3332 Hz frame rate at a spatial resolution of 128 × 128 pixels.

### 5.2. Moving Compensation for Modulation Patterns

The image blurring problem can be attenuated by moving compensation, which can be broadly divided into three types: mechanical compensation, optical compensation, and electronic compensation [61]. Mechanical compensation presents many difficulties in the design and control process of the device, and the quality of the reconstructed image is affected by the compensation accuracy. Electronic compensation, such as image restoration techniques, requires a large number of numerical operations, and the errors present in the computation process also affect the image quality. Optical compensation, however, can improve the resolution of the optical system and reduce the dependence on post-processing.

**Figure 11:**
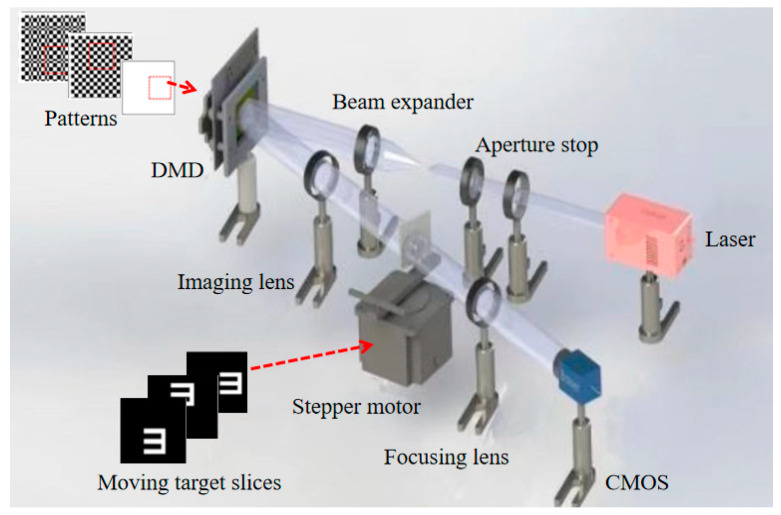
Tracking compensation method based on computational GI [63].

In 2014, Han et al. [37] successfully reconstructed a tangential moving target by translating the light intensity distribution on the reference optical path. In 2015, they [39] proposed a deblurring method based on speckle-resizing and speed retrieval. It obtains the velocity of a target with unknown motion parameters by retrieving the velocity, while the size of the patterns can be adjusted according to the nature of the different positions. It can overcome the effect of moving blur on the resolution of reconstructed images. In 2019, Sun et al. [62] proposed a moving estimation and quality enhancement scheme for a single image in dynamic SPI. When the motion state of the object is known, it is possible to build a model of the motion of the object. At this point, the object is assumed to be at rest, and then the modulated pattern is made equivalent to the motion along the opposite direction, resulting in a high-quality reconstructed image. In 2020, Yang et al. [63] proposed a tracking compensation method based on CGI; the schematic diagram is shown in Figure 11. This method allows accurate estimation of the target’s trajectory and the ability to move or rotate the illumination pattern preloaded on the DMD, compensating for angular velocities up to 5.45 μrad/s. It can eliminate moving blur and obtain high-quality reconstructed images with high signal-to-noise ratio. In 2022, Wu et al. [64] proposed a moving target tracking image method based on compressed perception and low-order moment estimation. It extracts the motion information of the target through low-order moments, gradually performs motion estimation and compensation during image processing, and finally reconstructs the image of the moving target using a compression-aware algorithm. It can effectively overcome moving blur and reduce the number of measurements required for each moving estimation.

At present, the research methods for ghost imaging of moving targets can be roughly divided into the above eight categories. In order to facilitate the understanding and comparison of different methods, we summarized the principles, advantages, and disadvantages of the above methods as well as the development direction in Table 1.

## 6. Challenges and Opportunities

In the past decade or so, with the research on moving target GI, the pace of GI engineering has been accelerated, and the image quality and efficiency of moving target GI have been significantly improved. At present, moving target GI is developing toward large field of view, long range, high resolution, and real-time. It is expected to be further applied in real-time image [59], objects classification [50], spatial remote sensing [39,51], unmanned driving [57], medical image [63], 3D image [59], and target tracking [53,60,63,65].

Although moving target GI has developed rapidly in recent years, the issues of blurred images and low real-time performance still exist, and how to improve the image performance of moving target GI is a current research hotspot, which the following three directions below, may give a breakthrough.

### 6.1. Stroboscopic Effect Introduced

At this stage, moving target GI is difficult for imaging high-speed objects or objects with high self-oscillation frequency; a stroboscopic instrument can effectively solve this problem. Stroboscopy, also known as transient light modulation (TLM) [66], refers to the light modulation caused by electrical modulation. An LED strobe light source can emit a specific frequency of light [67], and according to the estimated speed of the object in adjusting the light source strobe time, it can obtain the target object with the equal interval time displacement law. When the object moving speed is synchronized with the strobe source, because visual transient can make the object look relatively stationary, the quality improvement of the moving target GI can be achieved.

### 6.2. Modulation Pattern Combination

The key to GI is whether there is a rise and an attenuation of the optical field, and different modulation patterns have different effects on the rise and fall of the optical field. Hadamard patterns can improve the signal to noise ratio of the reconstructed image [68]; its algorithm is fast and the modulation matrix is generated quickly without data storage [69]. Wavelet patterns are better than Hadamard patterns at a low sampling rate [55], and the algorithm is efficient. The combination of Hadamard patterns and wavelet patterns are expected to achieve fast and efficient image reconstruction for moving targets at low sampling rates, improve the signal-to-noise ratio of the reconstructed images, and largely alleviate the image blurring problem caused by the relative moving between the object and the image system.

### 6.3. Reconstruction Algorithm Optimization and Innovation

The practical development of moving target GI requires the system to have real-time image capability. Neural networks with self-learning ability and self-adaptive capability can help GI achieve intelligence as early as possible, and GI can be realized at high speed in the face of moving targets in complex environments, which will greatly promote the improvement and development of unmanned technology. Combining GI reconstruction algorithms with more advanced neural network models, such as fast super-resolution convolutional neural network (FSRCNN) [70], is a useful way. FSRCNN extracts and reconstructs the features of the target image by a series of convolutional layers and nonlinear activation functions. It uses a jump connection technique which can reduce information loss while retaining more image details and can quickly and accurately achieve image reconstruction of the target object. This method is expected to improve the efficiency of the image system of the GI system and solve the problem of poor real-time GI of moving targets.

## 7. Conclusions

GI is a novel imaging technology, which can image the target object in an inaccessible environment, has characteristics of object image separation, advantages of high sensitivity, strong anti-interference ability, etc. It has broad application prospects in LiDAR imaging, remote sensing imaging, hyperspectral imaging, biomedicine, national defense, and military fields. GI is developing towards higher resolution, larger working distance, and larger field of view. Because of its flexible information acquisition and high detection sensitivity, it also brings new opportunities for moving object imaging in long distance, large field of view, and weak echo scenes.

In this paper, we reviewed and summarized the key techniques of moving target GI, introducing the existing research methods from the perspective of improving image speed and improving image quality. Among them, improving light source modulation can provide new solutions for in living microscopy, 3D imaging, and light detection and ranging. Due to the limitation of the algorithm, selecting the adaptive imaging region only images a single target in a background with uniform gray distribution. Selecting a suitable number of samples can play good roles in target tracking, living tissue imaging, medical imaging, and other fields. Estimating motion inter-frame information is appropriate for translational or rotating object imaging. Developing new reconstruction algorithms can be applied in rapid classification of flowing cells, assembly-line inspection, and aircraft classification in defense applications. The potential applications of tracking a target without image reconstruction include remote sensing imaging, biomedical imaging, and real-time tracking imaging. Designing new modulation patterns can be applied in remote sensing imaging and unmanned driving. Moving compensation for modulation patterns can play a great role in target tracking, remote sensing imaging, and medical diagnosis. At the same time, this paper also foresees the application areas and development directions of moving target GI, thereby providing references for further promoting the instantiation of GI applications.

## Figures and Tables

**Table 1:** Comparison of research methods.

Classification	Improve Imaging Speed						Improve Image Quality	
Core method	Improving light source modulation method	Selecting the adaptive imaging region	Selecting a suitable number of samples	Estimating motion inter-frame information	Developing new reconstruction algorithms	Tracking target without image reconstruction	Designing new modulation patterns	Moving compensation for modulation patterns
Principle	Develop a new LED array	Image the part of the area where the target is located, then place it at the location of the object in the scene	Select appropriate sampling number with the spatial sparsity of object	Divide the motion into several frames and estimate the information between them	Introduce another algorithm or neural networks into reconstruction algorithm	Obtain spatial information about the target object	Design the structure of patterns with the movement characteristics	Move patterns to make it remain relatively stationary with the object
Advantages	Improve the modulation speed of the light source	Reduce the number of patterns and have high numerical efficiency algorithm	Reduce sampling time, track and image multiple moving objects	Image moving objects in inaccessible environments	Have algorithms that require little computation	Have high speed detection and high efficiency calculation	Image objects in unknown motion states	Have a simple structure and does not require hardware compensation
Disadvantages	The power is unstable for a long time	It is only applicable to single target in the background of uniform gray distribution	Peripheral areas are not imaged properly	Objects moving too fast cannot be imaged	Algorithms related to deep learning require a lot of training	Unable to get an image of the target object	The imaging effect on rotating objects is not ideal	The specific motion of the object must be known
Development direction	Living microscopy, 3D imaging, light detection and ranging	Local imaging	Target tracking, living tissue imaging, medical imaging	Translational or rotating object imaging	Rapid classification of flowing cells, assembly-line inspection, aircraft classification in defense applications	Remote sensing imaging, biomedical imaging, Real-time tracking imaging	Remote sensing imaging, unmanned driving	Target tracking, remote sensing imaging, medical diagnosis

## Data Availability

The data presented in this study are available upon request from the corresponding author.
